# Supplementary material for: Patterns of Intron Gain and Loss in Fungi
Source: PLoS Biol. 2004 Nov 30;2(12):e422. doi: 10.1371/journal.pbio.0020422 (PMC532390; doi:10.1371/journal.pbio.0020422)
Supplement: Table S1 — Also available at http://genes.mit.edu/NielsenEtAl/. (4.3 MB ZIP). [file pbio.0020422.st001.zip › NielsenEtAl/html/687.html]

AN0254.1.NCU07468.1.MG09901.1.FG01227.1


```
 CLUSTAL W (1.82) Multiple Sequence Alignments - Introns Inserted


Sequence 1: MG09901.1	376 aa
Sequence 2: FG01227.1	375 aa
Sequence 3: NCU07468.1	375 aa
Sequence 4: AN0254.1	370 aa
Alignment Length: 377 aa
Number Identitical Residues: 168 aa
Alignment Score (without introns) 9583


MG09901.1 	MAEK-PSVLIIGGLG~YIGRFLALHIHKNGLASEVRVVDKVLPQLAWLAPEFKDACAGDK
NCU07468.1	MAEK-PSVLIIGGLG~YIGRFLALHIHKNNLASEVRIVDKVLPQLAWLAPEFEEACAGPK
FG01227.1 	MAEK-PSVLIIGGLG~YIGRFLALHIHQNDLASDVRLVDKVLPQLAWLAPEFSEACSQDK
AN0254.1  	MTDKKPSVLIIGGLG1FIGRHLALYIHENNLASEVRLVDKVLPQLAWLAPEFEEACSKDK
          	*::*.********** :***.***:**:*.***:**:***************.:**:  *

MG09901.1 	FVQADASRKE1SMARIFDRPDGKEWDYVFNCGGETRYSQEDEVYKLRSLGLSLALGNEAA
NCU07468.1	FMQADASREQ1SLQRIFDRSNGKQWDYVFNCGGETRYSQEEEVYKLRSLELSLAVGREAA
FG01227.1 	FMQADASRPE1GLARIFDRPDGKQWDYVFNCGGETRYSQEDEVYKLRSLNLSIAVGKEAA
AN0254.1  	FVQADASREQ1HFPRIFDRANGEQFDYVINCGGETRYSQPDDVYEARVYNLSVALGREVA
          	*:****** :  : *****.:*:::***:********** ::**: *   **:*:*.*.*

MG09901.1 	KRKIKVFVELSTGMVYKPDSQPSKEGDKQKPWSKIAVFKQQAEEQLAKIEG2LNLVIARL
NCU07468.1	KRGVKCFIELSTGMVYKPDSAPSKEQDKLKPWSKIAVFKLQAEEELAKIPG2LNLAIVRL
FG01227.1 	KRGVKAFFELSTGMVYKSDSSPSKEVDKLKPWNRIAVFKLQAEEELSKIEG2LNLIIVRL
AN0254.1  	RRGIKSFVECSTAHVYKGGSTPRKEGDKIQAWHKLAKWKLKANDELMKTPG~LHHVVLRL
          	:* :* *.* **. *** .* * ** ** :.* ::* :* :*:::* *  * *:  : **

MG09901.1 	AHVYGPYASQWVATALCMARVYKEKDEEMKWLWTKDLRTNTVHISDVVRALWDMASWYDA
NCU07468.1	SHVYGPYASQWVATALCMARVYQALGSEMKWLWTKNLCTNTVHIHDVTRALWQVAEWYAA
FG01227.1 	PHVYGPYASQWVATALCMARTYQHLEDEMKWLWTKDLRTNTAHIDDVTRALWDIAAWYDA
AN0254.1  	PNVFGEYESGYFAMGICLARVYLDMQKDLELLYSKDLKTNTLYVKDAASALWKAAEWRYS
          	.:*:* * * :.* .:*:**.*    .::: *::*:* *** :: *.. ***. * *  :

MG09901.1 	GKKGWDNKTMGEVATFNVVDKGVTTQGAMAELIGDIFGIQTGFQGQLISTFARLNMDSVV
NCU07468.1	GKAKWDAAKMGPTPTFNVVDKGRTTQGIMADLIGDVFGIKTGFQGTIISNFAKIHLHDVV
FG01227.1 	GKAKWDEASMGKVPIFNIVDEGATSQGTIATIIGDIFKIETGFQGQLISTFARLNLDSVV
AN0254.1  	APTDGSAPIL-----FNVVDHNDTRQQHVADALTEVFGLKCSFLGSLASQFAKLNLDDVV
          	.    .   :     **:**.. * *  :*  : ::* :: .* * : * **::::..**

MG09901.1 	DDVNDNMLGPWAELLEQAGIVRPGPLTPFMEKELLKDTDLSMDGTRLETVLGFQYEKPKI
NCU07468.1	EDVNDELLGPWAGLLEDAGITKPGPLTPFMEAELLKDTDLSMDGSRLEELLGFKYEKPQI
FG01227.1 	DDVNDELLGPWADLLADAGITRPGPLTPFMEKELLKDTDLSMDGTRLKTLLGFEYKKPTL
AN0254.1  	DDMNEVSLQGWSDLIEEKGITRPGPISPFIERDVLQDQDMSLDGTLFETTTGWKPTR-NF
          	:*:*:  *  *: *: : **.:***::**:* ::*:* *:*:**: ::   *::  :  :

MG09901.1 	TKELLEEVIESYKNMNWWPA
NCU07468.1	NKELLQEVIESYKRMKWWP-
FG01227.1 	TKELIEEVIESYKKMKWWP-
AN0254.1  	GPDSVRDMVESYKRMGWWP-
          	  : :.:::****.* ***
```
